# Hypoxic BMSC-derived exosomal miR-652-3p promotes proliferation and metastasis of hepatocarcinoma cancer cells via targeting TNRC6A

**DOI:** 10.18632/aging.205025

**Published:** 2023-11-16

**Authors:** Mei Li, Pengtao Zhai, Xudong Mu, Juanrong Song, Huilin Zhang, Juan Su

**Affiliations:** 1 Department of Minimally Invasive, Shaanxi Cancer Hospital, Xi’an 710061, Shaanxi, China; 2 Digestive Endoscopy Treatment Center, Xi’an International Medical Center Hospital, Gaoxin, Xi’an 710100, Shaanxi, China; 3 Department of Gastroenterology, Xi’an International Medical Center Hospital, Gaoxin, Xi’an 710100, Shaanxi, China

**Keywords:** hypoxic BMSCs, exosome, miR-652-3p, TNRC6A

## Abstract

Cancer microenvironment plays an important role in the proliferation and metastasis of hepatocarcinoma cancer cells (HCC). Exosomes from bone marrow-derived mesenchymal stem cells (BMSCs) are a component of the cancer microenvironment. In this study, we reveal that miRNA-652-3P from BMSC-derived exosomes promotes proliferation and metastasis in HCC. The ability of cancer proliferation, migration and invasion can be evaluated after co-culture by CCK-8, wound healing and transwell assay. Isolated exosomes were identified by transmission electron microscopy (TEM) and the biomarkers of the purified exosomes were showed in West-blotting (WB). MiR-652-3p was detected in the HepG2 and 7721 after co-culturing with exosome derived from BMSCs under different conditions. Target authentication was performed by a luciferase reporter assay to confirm the presumptive target of miR-652-3p. After overexpressing miR-652-3p, the mRNA and protein expression level of TNRC6A in HCC was examined by q-PCR and WB. Further, we observed greater miR-652-3p upregulation in hypoxic BMSCs-exosomes than in normal- exosomes. In addition, a miR-652-3p inhibitor attenuates the proliferation and metastasis of HCC cells after co-culturing with BMSCs. Our data demonstrate that hypoxic BMSCs-derived exosomal miR-652-3p promotes proliferation in HCC cells by inhibiting TNRC6A. The BMSCs-derived exosomal miR-652-3p may help find patient-targeted therapies in hepatocarcinoma cancer.

## INTRODUCTION

The tumor microenvironment induced by interactions between hepatocarcinoma cancer cells (HCC) and bone marrow-derived mesenchymal stem cells (BMSCs) is critical for HCC proliferation and metastasis, HCC microenvironment consists of stromal cells, extracellular and HCC components [1]. Stromal cells that contribute to HCC proliferation and metastasis are mainly BMSCs and others immune cell. Communication interactions between HCC and BMSCs depend on exosomes [2]. Some experimental studies have revealed that BMSCs release specialized exosomes, which contribute to tumor progression and metastasis [3].

Exosomes are one of the smallest extracellular vesicles (EVs) released from all cell types, approximately 40–150 nm size [4]. Exosomes are composed of a lipid bilayer like cell membranes, and contain all known molecular constituents of a cell, such as DNA, proteins, and RNA [5]. Previous studies show that around 1300 different RNAs are enclosed in exosomes by microarray assessment, shuttle RNA of exosomes, such as MicroRNAs (miRNAs), which are delivered into recipient cells, some of which have been proven to be functional [6]. Some miRNAs contained in stem cell-derived exosomes could be a promising metastasis and proliferation in the context of hypoxic conditions [7, 8].

MiRNAs are non-coding and endogenous RNA, approximately 22 nt, regulating gene expression post-transcriptionally [9]. Myriad studies were proved function of miRNAs in the pathogenesis of cancer, such as a seminal study showed that miR-15a/16-1 cluster is frequently deleted in chronic lymphocytic leukemia (CLL), implicating function of these miRNAs in CLL maybe as tumor suppressors [10]. Several previous studies have been demonstrated that function of many miRNAs as tumor suppressor in the majority of cancers profiled by far, but function of other miRNAs as oncogenous-miR in the most of cancer malignancies profiled, such as abnormal expression of miR-155 was found in Reed-Sternberg cells of Hodgkin’s Lymphoma [11]. MicroRNAs can post-transcriptionally mediate gene silencing mRNA of target genes, which by targeting specific sites in the 3’untranslated region, previous study demonstrated that miR-26a-5p promote theca cell proliferation by regulates TNRC6A expression [12].

TNRC6A, also known as GW182, protein of TNRC6A have multiple Glycine- Tryptophan (GW) repeats in the N-terminal and molecular weight are a 182 kDa [13]. In vertebrate, the family of GW182 including three—TNRC6A, 6B, and 6C. GW182 family proteins play pivotal role in miRNA- regulatory gene suppression [14]. Li Kang and colleagues demonstrated the analyzed the relationship between miR-26a-5p and TNRC6A in chicken ovary and ovarian follicles, further proved function of metastasis and proliferation of miR-26a-5p and TNRC6A in chicken ovarian theca cell [15].

Therefore, in this work, we first demonstrated hypoxic BMSC-derived exosomal miR-652-3p promotes metastasis and proliferation of hepatocarcinoma tumor cells via targeting TNRC6A. According to this study, we hope to provide some new ways in the comprehending and therapy of HCC.

## RESULTS

### Hypoxic BMSCs co-culture promotes HCC cells proliferation and metastasis *in vitro*

**Figure 1:**
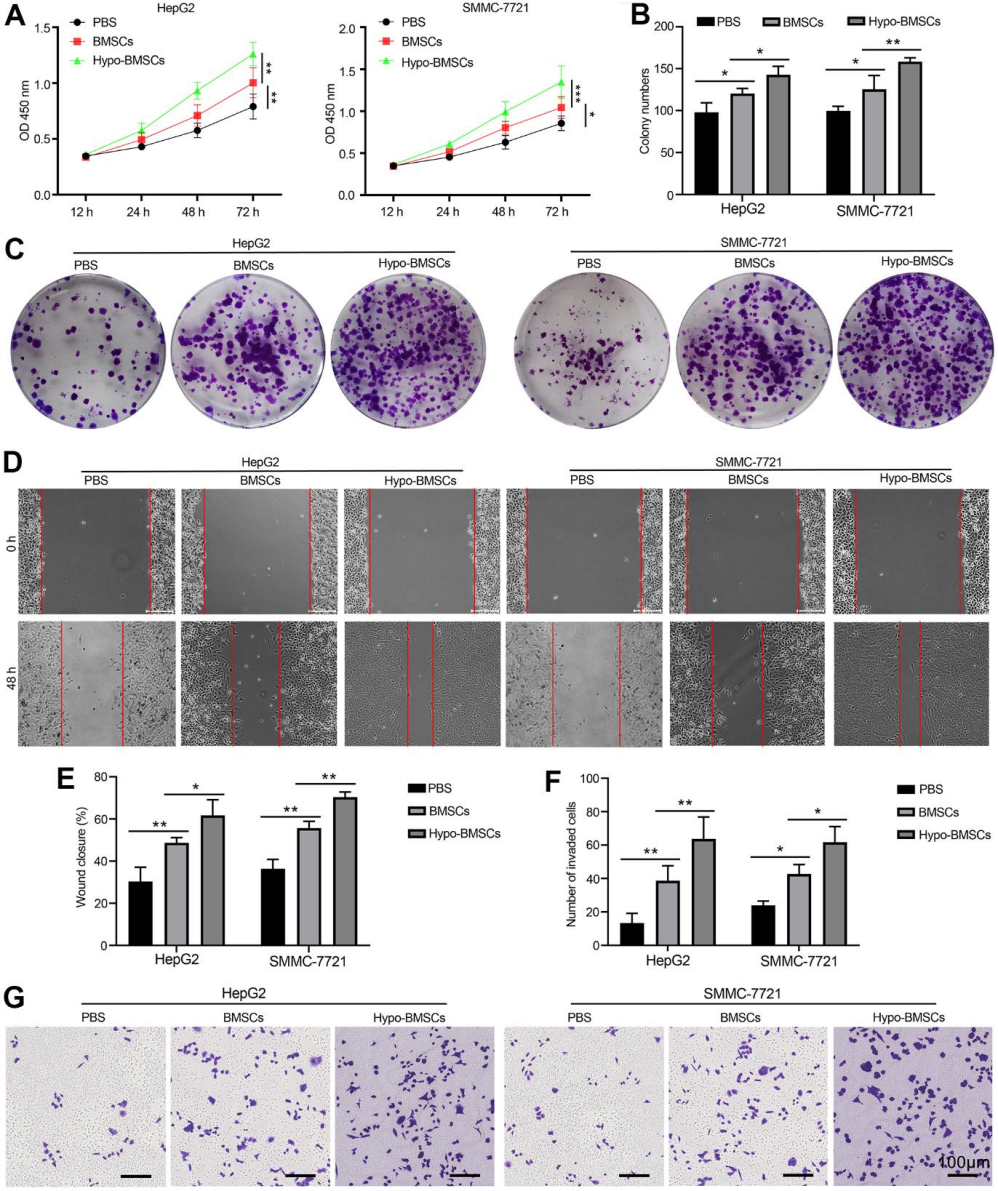
**Hypoxic BMSCs co-culture promotes HCC cells proliferation and metastasis *in vitro*.** (**A**) Proliferation of HepG2 and SMMC-7721 cells determined by CCK-8 after co-culturing with BMSCs or hypo-BMSCs, Data were presented as the mean ± SD, and analyzed with Student’s t-test. *P < 0.05; ** P < 0.01. (**B**) The numbers of colony were counted from six fields of view in each group. Data were presented as the mean ± SD, and analyzed with Student’s t-test. *P < 0.05; ** < 0.01. (**C**) Colony formation assays showed that the proliferation rate was increased in HepG2 and SMMC-7721 cells after co-culturing with BMSCs or hypo-BMSCs. (**D**) Cell migration was measured by wound healing assay. The increased migration capability induced by hypoxic BMSC-secreted exosomes. (**E**) The distance of migration was measured from six fields of view in each group. Data were presented as the mean ± SD, and analyzed with Student’s t-test. *P < 0.05; ** P < 0.01. (**F**) The numbers of dot violet were counted from six fields of view in each group. Data were presented as the mean ± SD, and analyzed with Student’s t-test. *P < 0.05; ** P < 0.01. (**G**) Cell invasion were measured by transwell assays. HepG2 and SMMC-7721 cells co-culturing with BMSCs or hypo-BMSCs for 48 h. Cells that invaded to the bottom surface were stained with crystal violet and observed by light microscopy (magnification, 10×).

To explore the potential interactions between HCC and BMSCs, we first developed co-culture model. HepG2 cells and SMMC-7721 cancer cells line perform many differentiated hepatic functions and they with high proliferation rates, proliferation of HepG2 and SMMC-7721 cells as measured by CCK8 after co-culturing with BMSCs or hypo-BMSCs in 12h, 24h, 48h and 72h. As showed in Figure 1A, the proliferation levels are higher HepG2 and SMMC-7721 cells co-culture with hypo-BMSCs than BMSCs and control group, the result demonstrated that BMSCs or hypo-BMSCs co-culture promote HCC cells proliferation. Further, in colony formation assay, histogram (Figure 1B) shown that HepG2 and SMMC-7721 cells co-culture with hypo-BMSCs or BMSCs formed markedly more colonies than control group (Figure 1C). Next, the migration and invasion ability of cells were measured by wound healing (Figure 1D) and transwell assay (Figure 1G), respectively, after co-culturing with BMSCs or hypo-BMSCs. The data of histogram (Figure 1E, 1F) revealed that the migration or invasion ability of HepG2 and SMMC-7721 cells markedly increased in 48h compared with control.

### MiR-652-3p is upregulated in hyoxic BMSCs-derived exosome and can be transferred to HCC cells

**Figure 2:**
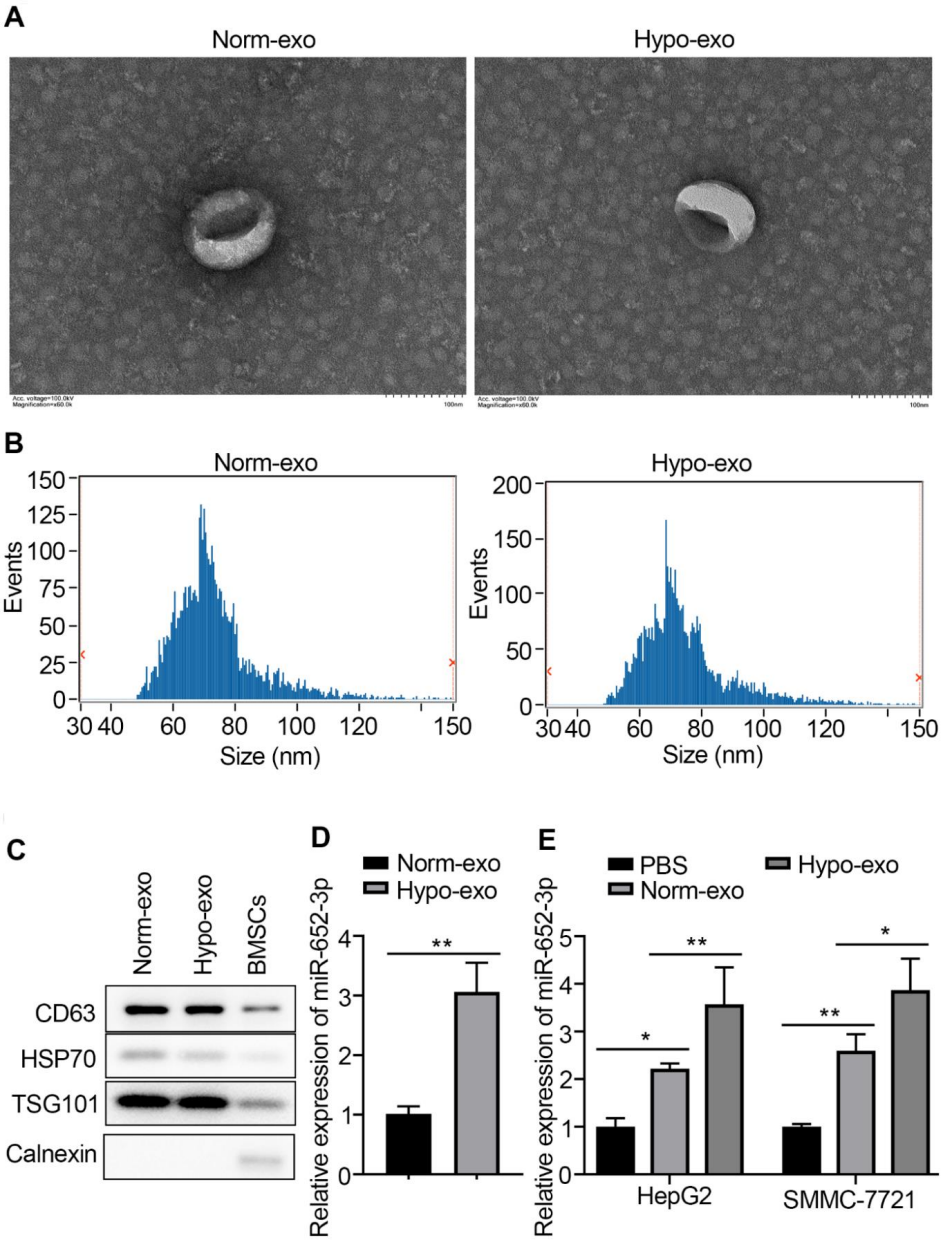
**MiR-652-3p is upregulated in hyoxic BMSCs-derived exosome and can be transferred to HCC cells.** (**A**) Transmission electron microscopy (TMB) showed the representative image of BMSCs or hypo-BMSCs derived exosome. (**B**) The particle diameter of the purified exosomes was showed in histogram. (**C**) Exosomal markers were detected by West blotting in BMSCs derived exosome and BMSCs cells. (**D**) Expression of miR-652-3p was detected in exosome derived from BMSCs under different conditions using qRT-PCR. Data were presented as the mean ± SD, and analyzed with Student’s t-test; ** P < 0.01, compared with the indicated controls. (**E**) Expression of miR-652-3p was detected in HepG2 and SMMC-7721 cells after co-culturing with exosome derived from BMSCs under different conditions. Data were presented as the mean ± SD, and analyzed with Student’s t-test. *P < 0.05; ** P < 0.01, compared with the indicated controls.

More and more evidence demonstrate that exosomes play an import role in facilitating tumorigenesis by regulating cancer microenvironment [16]. To elucidate underlying mechanism, we first isolate exosomes by procedures involving ultracentrifugation and quantify exosomes accurately by used the transmission electron microscopy (TEM) [17]. Exosomes isolated and purify from freshly-harvested supernatant of BMSCs or hypo-BMSCs culture, were evaluated by Western blots, NanoSight and TEM (Figure 2A). The ultracentrifuged, resuspended BMSC-derived exosomal appear in TEM has thus a characteristic well defined membrane-bound vesicles. Test of exosomes isolated from supernatant of hypo-BMSCs or normal cell cultural in the NanoSight instrument gave a broad peak corresponding to the mean particle size of 70 nm with the range of 50–150 nm (Figure 2B). Western blots were performed to determine the biomarkers of exosome (markers including CD63, HSP70, TSG101) in isolated BMSCs derived exosomes (Figure 2C). Interestingly, expression of miR-652-3p was detected in exosome derived from BMSCs under different conditions using qRT-PCR (Figure 2D). Further, high expression of miR-652-3p also was detected in HepG2 and SMMC-7721 after co-culturing with exosome derived from BMSCs under different conditions (Figure 2E).

### Inhibit miR-652-3p attenuates the proliferation and metastasis of HCC cells after co-culturing with BMSCs

**Figure 3:**
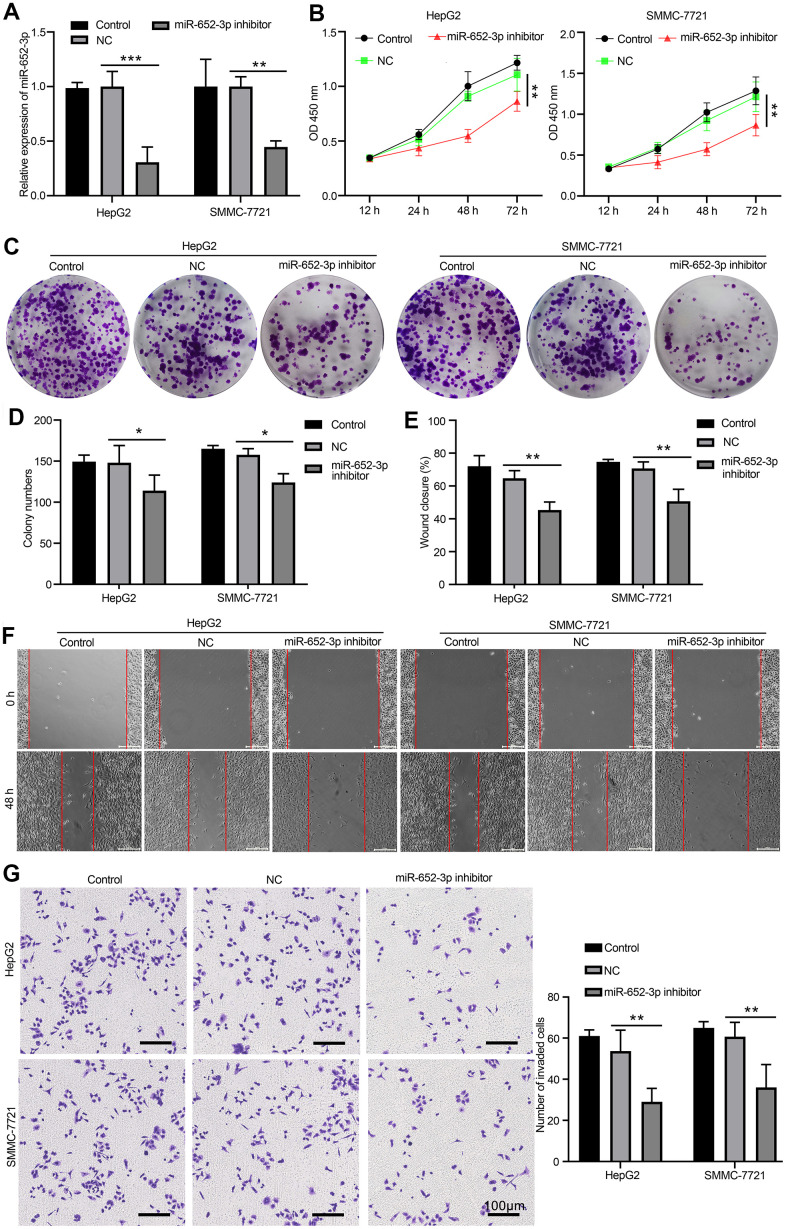
**Inhibit miR-652-3p attenuates the proliferation and metastasis of HCC cells after co-culturing with BMSCs.** (**A**) Expression of miR-652-3p was detected in HepG2 and SMMC-7721 cells co-culturing with BMSCs or hypo-BMSCs by using qPCR assay. Data were presented as the mean ± SD, and analyzed with Student’s t-test. **P < 0.01; *** P < 0.001, compared with the indicated controls. (**B**) Cell growth curves in HepG2 and SMMC-7721 cells transfected with different combinations. Data were presented as the mean ± SD, and analyzed with Student’s t-test. *P < 0.05; ** P < 0.01, compared with the indicated NC controls. (**C**) Colony formation assays showed that the proliferation rate was decreased in HepG2 and SMMC-7721 cells after treat isolated exosome hypo-BMSCs-derived with miR-652-3p inhibitor. (**D**) The numbers of colony were counted from six fields of view in each group. Data were presented as the mean ± SD, and analyzed with Student’s t-test. *P < 0.05. (**E**) The distance of migration was measured from six fields of view in each group. Data were presented as the mean ± SD, and analyzed with Student’s t-test. *P < 0.05; ** P < 0.01. (**F**) The numbers of dot violet were counted from six fields of view in each group. Data were presented as the mean ± SD, and analyzed with Student’s t-test. *P < 0.05; ** P < 0.01. (**G**) Cell invasion were measured by transwell assays. HepG2 and SMMC-7721 cells co-culturing with isolated exosome hypo-BMSCs-derived with miR-652-3p inhibitor treat for 48 h. Cells that invaded to the bottom surface were stained with crystal violet and observed by light microscopy (magnification, 100×).

To further investigate the function underlying of miR-652-3p to promote proliferation and metastasis of HCC cells after co-culturing with BMSCs. After BMSCs by hypoxia condition treatment, exosome was isolated with hypo-BMSCs transfected with NC or miR-652-3p inhibitor, next HepG2 and SMMC-7721 cells were co-cultured with above exosome. The expression of miR-652-3p in HCC cells determined by qRT-PCR, the qRT-PCR analyses revealed that the expression of miR-652-3p is markedly reduced in miR-652-3p inhibitor group compared with NC and control (Figure 3A). As showed in Figure 3B, the proliferation levels of HepG2 and SMMC-7721 cells with miR-652-3p inhibitor are markedly lower than NC and control. After co-cultured with above exosome, next examination different ability of HepG2 and SMMC-7721 cells, such as Colony formation (Figure 3C, 3D), migration (Figure 3E, 3F) and invasion (Figure 3G), we confirmed that the proliferation and metastasis levels of HepG2 and SMMC-7721 cells with miR-652-3p inhibitor are markedly weaker than NC and control. In addition, the results indicate that overexpression of miR-652-3p inhibitor attenuates the proliferation and metastasis of HepG2 and SMMC-7721 cells after co-culturing with hypo-BMSCs.

### TNRC6A is a direct target of miR-652-3p in human HCC cells

**Figure 4:**
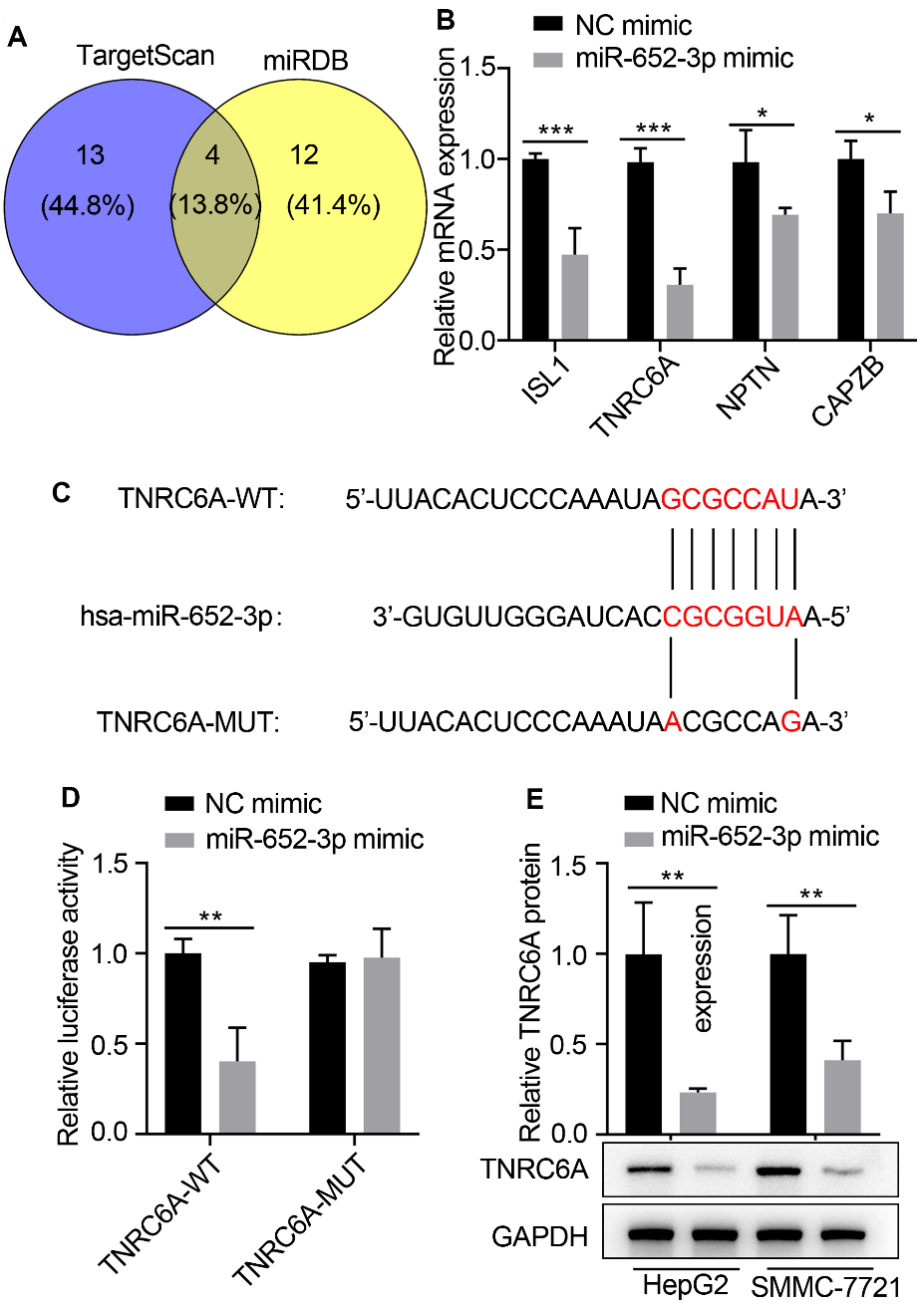
**TNRC6A is a direct target of miR-652-3p in human HCC cells.** (**A**) The underlying targets of miR-652-3p were predicted using TargetScan and miRDB databases. (**B**) Expression of target genes in HepG2 cells determined by qRT-PCR after transfecting with miR-652-3p mimic. (**C**) Scheme and sequence of the intact miR-652-3p, TNRC6A (Wt) and its mutant (Mut). Computer prediction of miR-652-3p binding sites in the 3’UTR of human TNRC6A gene. (**D**) SMMC-7721 cells were co-transfected with miR-652-3p and WT or MUT 3’UTR of TNRC6A. Data were presented as the mean ± SD, and analyzed with Student’s t-test. ** < 0.01; compared with the indicated NC mimic controls. (**E**) Protein level of TNRC6A was detected by WB in HepG2 and SMMC-7721 cells transfected with NC mimic and miR-652-3p. GAPDH was also detected as a loading control. Data were presented as the mean ± SD, and analyzed with Student’s t-test; ** P < 0.01.

To further investigate the molecular mechanisms underlying of miR-652-3p to promote proliferation and metastasis of HepG2 and SMMC-7721 cells after co-culturing with BMSCs, we used bioinformatics analysis methods such as TargetScan and miRDB to predict the target genes based on the consensus binding sites of miR-652-3p, eventually TargetScan and miRDB returns 4 predicted common target genes (Figure 4A). Next expression of target genes in HepG2 cells determined by qRT-PCR after transfecting with miR-652-3p mimic, the qRT-PCR result showed that four gene largely increase, which including ISL1, TNRC6A, NPTN and CAPZB (Figure 4B). TNRC6A was predicted to be a directly target of miR-652-3p. To confirm whether TNRC6A was a direct target of miR-652-3p in HCC, next the mutant of TNRC6A was constructed (Figure 4C)and perform dual luciferase assays, Our data present that the luciferase activities were notably deceased after co-transfection of wild type TNRC6A 3’ UTR and miR-652-3p mimics. However, remained largely unchanged both after co-transfection of miR-652-3p mimics and mutated TNRC6A 3’ UTR, or co-transfection of NC and wild type TNRC6A 3’ UTR in HCC, respectively (Figure 4D). These results implied the direct binding of miR-652-3p to the 3’UTR of TNRC6A. In addition, TNRC6A mRNA level was significantly decreased in HepG2 and SMMC-7721 cells transfected with miR-652-3p mimic in comparison to the cells transfected with NC (Figure 4E). Moreover, our WB assays also demonstrated markedly decreased protein levels of TNRC6A in HepG2 and SMMC-7721 cells when transfected with miR-652-3p mimic (Figure 4E). Therefore, above results indicate that TNRC6A is a direct downstream target of miR-652-3p in HepG2 and SMMC-7721 cells.

### Overexpression of miR-652-3p aborted the inhibitive effects of TNRC6A on the proliferation and metastasis of HCC cells

**Figure 5:**
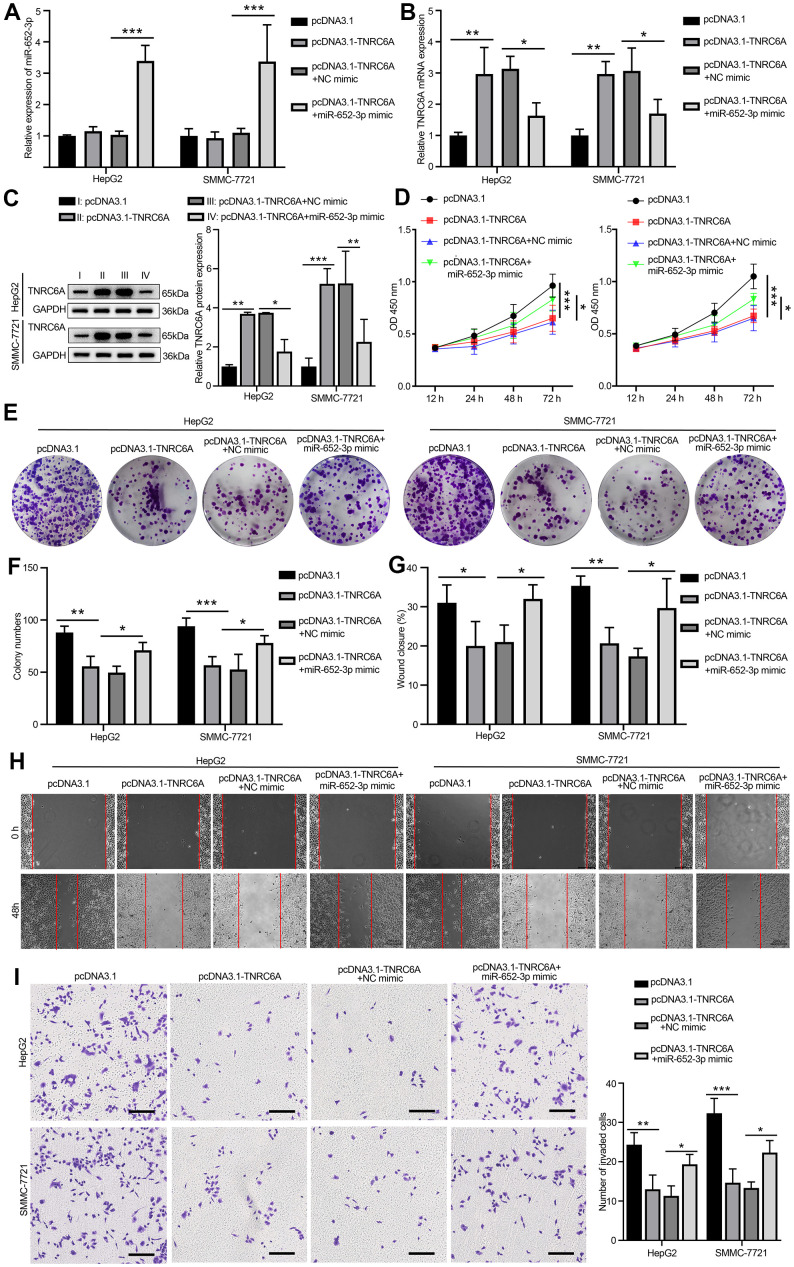
**Overexpression of miR-652-3p aborted the inhibitive effects of TNRC6A on the proliferation and metastasis of HCC cells.** (**A**) The expression of miR-652-3p inHepG2 and SMMC-7721 cells determined by qRT-PCR, Cells were treated with four different means: pcDNA3.1 group, pcDNA3.1-TNRC6A group, co-transfection pcDNA3.1-TNRC6A and NC mimics and co-transfection pcDNA3.1-TNRC6A and miR-652-3p mimic group. Data were presented as the mean ± SD, and analyzed with Student’s t-test *** P < 0.001 (**B**) The mRNA expression of TNRC6A in HepG2 and SMMC-7721 cells determined by qRT-PCR. Data were presented as the mean ± SD, and analyzed with Student’s t-test *P < 0.05; ** P < 0.01. (**C**) The protein expression of TNRC6A in HepG2 and SMMC-7721 cells determined by WB. Histogram show the protein expression of TNRC6A in HepG2 and SMMC-7721 cells. Data were presented as the mean ± SD, and analyzed with Student’s t-test *P < 0.05; **P < 0.01, ***P < 0.001. (**D**) Proliferation of HepG2 and SMMC-7721 cells determined by CCK-8 after treating with different ways, Data were presented as the mean ± SD, and analyzed with Student’s t-test. *P < 0.05 *** P < 0.001. (**E**) Colony formation assay of HepG2 and SMMC-7721 cells after treating with different ways (**F**) Histogram show the amount of colony in HepG2 and SMMC-7721 cells. Data were presented as the mean ± SD, and analyzed with Student’s t-test *P < 0.05; ** P < 0.01 *** P < 0.001. (**G**) Histogram show the distance of cellular migratory in HepG2 and SMMC-7721 cells. Data were presented as the mean ± SD, and analyzed with Student’s t-test *P < 0.05; ** P < 0.01. (**H**) Migration of HepG2 and SMMC-7721 cells determined by wound healing. (**I**) Invasion of HepG2 and SMMC-7721 cells determined by transwell. Cells that invaded to the bottom surface were stained with crystal violet and observed by light microscopy (magnification, 100×). Histogram show the capability of cellular invasion in HepG2 and SMMC-7721 cells. Data were presented as the mean ± SD, and analyzed with Student’s t-test *P < 0.05; ** P < 0.01 *** P < 0.001.

To further substantiate the target relationship between miR-652-3p and TNRC6A in HepG2 and SMMC-7721 cells. Cells were treated with four different means: pcDNA3.1 group, pcDNA3.1-TNRC6A group, co-transfection pcDNA3.1-TNRC6A and NC mimics and co-transfection pcDNA3.1-TNRC6A and miR-652-3p mimic group, next the miR-652-3p expressions in HepG2 and SMMC-7721 cells were determined by quantitative RT-PCR. The quantitative RT-PCR assay results demonstrated that TNRC6A over-expression significantly enhanced the mRNA level of TNRC6A and markedly inhibited the miR-652-3p level of HepG2 and SMMC-7721 cells (Figure 5A, 5B). And the mRNA level of TNRC6A was adverse with the miR-652-3p (Figure 5B). According to the WB assay, the protein of TNRC6A markedly higher in overexpression group compare with co-transfection pcDNA3.1-TNRC6A and miR-652-3p mimic group (Figure 5C). Next the proliferation of HepG2 and SMMC-7721 cells determinate by CCK-8 after transfection with different matter in 12h, 24h, 48h and 72h, the result showed that the proliferation of HepG2 and SMMC-7721 cells in overexpression TNRC6A group significantly decrease compare with other group, while overexpression of miR-652-3p aborted the inhibitive effects of TNRC6A on the proliferation of HepG2 and SMMC-7721 cells (Figure 5D). A similar conclusion emerged separately in colony formation assay (Figure 5E), wound healing (Figure 5H) and transwell (Figure 5I) assay. As show in histograms (Figure 5F) and (Figure 5G) that the colony formation, migration and invasion capacity of HepG2 and SMMC-7721 cells in overexpression TNRC6A group significantly decrease compare with other group, while overexpression of miR-652-3p aborted the inhibitive effects of TNRC6A on the proliferation of HepG2 and SMMC-7721 cells. According to these data demonstrated that miR-652-3p abrogates TNRC6A on the proliferation and metastasis of HepG2 and SMMC-7721 cells.

## DISCUSSION

Various studies have reported that tumor microenvironment plays an important function in the proliferation and metastasis of cancer cells [18]. Cancer microenvironment consists of various cellular, cellular secretion and extracellular components, such as BMSCs and BMSCs-derived exosomal [19]. More and more research has been focused on the tumor microenvironment of cancer and its function [20, 21]. In this article, after BMSCs or hypo-BMSCs co-culture with HepG2 and SMMC-7721, to promote HepG2 and SMMC-7721 cells proliferation and metastasis *in vitro*, the result of above are similar to previous report [22].

Exosome as one of cellular secretion have been attributed for their roles in proliferation and metastasis of cancer [23]. Due to their low immunogenicity, long half-life in the circulation and the nanoparticles characteristic of exosomes, it as the major mediators in cell–cell communication [24]. The size of exosome approximately 50–150 nm and exosomes contain various components of host cells, such as proteins, nucleic acids, RNA (mRNA, miRNA and LncRNA) and DNA [25]. Exosomes derived from different cell types have different characteristics, such as biomarker of T cells or tumor cells derived exosomes including CD3 [25]. Our data revealed that the size of BMSCs-derived exosomal approximately 50–150 nm and biomarker including CD63, HSP70, TSG101.

Various mechanisms have been reported that miRNA promote metastasis and proliferation of cancer cells via exosome way [26]. Previous evidence suggests that anomalous expression of miRNAs in various cellular of tumor microenvironment can promote cancer cells metastasis and proliferation by means of exosome pathway [27]. Previous studies demonstrated that exosomal miRNAs and miRNA dysregulation mediate various cancer cells proliferation, migration and invasion. Such as colonization and invasion potential of tumor cells is enhanced when miR-200 from exosomes of invasive tumor cells is transferred to less invasive tumor cells [28]. Several recent studies demonstrated that Hypoxic BMSC-derived exosomal miRNAs play various import function in cell communication, such as miR-214 derived from BMSCs regulate oxidative damage in cardiac stem cells [29], promote proliferation of lung cancer cells Epithelial-mesenchymal transition [30], miR-101-loaded exosomes secreted by BMSCs facilitating osteogenic differentiation [31], BMSC-derived exosomal miR-340 inhibits myeloma-related angiogenesis [31]. However, the correlation between Hypoxic BMSC-derived exosomal miRNAs and hepatocarcinoma cancer cells remains unclear.

In this research, we found that Hypoxic BMSC-derived exosomal miR-652-3p promotes proliferation and metastasis of hepatocarcinoma cancer cells. Emerging evidence has demonstrated miR-652-3p play various function in different cell, such as effect on apoptosis and drug sensitivity in pediatric acute lymphoblastic leukemia [32], induces Epithelial-mesenchymal transition in PC3 prostate cancer cells [33], promotes proliferation and metastasis in non-small cell lung cancer [34]. Further mechanistic investigations provided evidence highlighting the effects of miR-652-3p promotes proliferation and metastasis in HCCs was achieved through targeting and restricting TNRC6A expression. TNRC6A, also name GW182, major function is an Argonaute-navigator protein for microRNA-mediated gene silencing [35], recent evidence demonstrated that miR-26a-5p facilitates theca cell metastasis in chicken ovarian follicles via regulates TNRC6A expression [36], TNRC6A-mediated miRNA function is development of yolk sac endoderm [37]. However, we first discover that miR-652-3p from exosomal of Hypoxic BMSC-derived and TNRC6A promotes proliferation and metastasis in HCCs.

In a summary, the mechanistic of exosomal miR-652-3p of our found in the tumor microenvironment may help provide new diagnostic and therapeutic target for HCCs.

## MATERIALS AND METHODS

### Cell culture and co-culture system

Two liver tumor cell lines: smmc-7721 and hepG2 were got from The Cell Bank of Type Culture Collection of the Chinese Academy of Sciences. Cells were developed in Dulbecco’s modified Eagle’s medium (DMEM) (HyClone; Cytiva, USA) and added 1% penicillin-streptomycin (Thermo Fisher Scientific, Inc.), with 10% fetal bovine serum (FBS, HyClone; Cytiva, USA). Bone marrow derived stem cells (BMSCs) were obtained from Shanghai Fusheng Biotechnology (Shanghai, China). After passaging for one time, the primary BMSCs were cryopreserved in liquid nitrogen using a cryopreservation medium (EXINNO). When recovered, the BMSCs were cultured in DMEM (Sigma, USA). All cells were normally cultured in a cell incubator at 37° C with 5% CO_2_. For hypoxic culture, cells were cultured in medium at 37° C with 5% CO_2_ and 1% oxygen in nitrogen in a 3-gas incubator.

Transwell chamber (4.0-μm, Corning, USA) was used for the co-culture system. Briefly, ~ 3.0×10^4^ of hepG2 and smmc-7721 were planted in 24-well-plate with 500 μl medium and 2.0×10^5^ of BMSCs were seeded onto the transwell chamber with 200 μl medium. Six hours after seeding, transwell chamber was inserted into 24-well plate and co-culturing at 37° C with 5% CO_2_. Fresh media were replaced every 2 days, and hepG2 and smmc-7721 culture single was considered as the control. On the day 5 after co-culture, the hepG2 and smmc-7721 cells were harvested.

### Cell transfection

The miR-652-3pmimic (5ʹ-AAUGGCGCCACUAGGGUUGUG-3ʹ), mimic negative control (mimic NC, cat. no. miR1N0000001-1-10), miR-652-30 inhibitor (5ʹ-CACAACCCUAGUGGCGCCAUU-3ʹ), and inhibitor NC (cat. no. miR2N0000001-1-10) were obtained from RIBOBIO biotechnology company (Guangzhou, China). Meanwhile, pcDNA3.1 and pcDNA3.1-TNRC6A were gained from GenePharma (Shanghai, China). For transfection, hepG2, smmc-7721, or BMSCs were planted in a six-well-plate at 37° C for at least 24 h with 70% confluency. Then, the Lipofectamine® 3000 kit (Thermo Fisher Scientific, Inc.) was used to transfect cells with indicated plasmids for 48 h.

### Isolation and characterization of exosome

For exosome collection, cells were cultivated in exosome-free FBS (Gibco, USA) at 37° C under normoxic or hypoxic condition. After 48h culturing, the supernatants of cells were collected and exosome in the supernatants were isolated using differential centrifugation. Briefly, cell supernatants were firstly centrifuged at 1,000×g for 15 min to remove intact cells. Next, the supernatants were centrifuged a 10,000×g for 30 min in turns to remove debris and large vesicles. Then, the supernatants were harvested followed by 0.22-μm filtration (Millex, Germany). After this, the supernatants were centrifuged at 100,000 ×g for 70 min for collect pallet exosome. Subsequently, exosome pellet was washed with PBS, harvested by 100,000 ×g centrifugation for 1 h, and assessed via transmission electron microscopy (TEM; JEM-1400, JEOL, Japan) and nanoparticle tracking analysis (NTA; Zeta View, Particle Metrix, DE). The protein concentration of exosome was qualified by the Bradford assay (Sangon, Shanghai, China) and exosomes were confirmed by specific markers CD63, HSP70, TSG101, and Calnexin were determined by western blotting.

### Cell counting kit-8 (CCK-8) assay

The CCK-8 kit (AccuRef Scientific, Xi’an, China) was utilized to analyze cell proliferation. HepG2 or SMMC-7721 cells with different treatments were cultured in a 96-well plate (2000 cells/well). Later, 10 μl of the Cell Counting Kit-8 reagent was added to each well and incubated for 2 hours at 37° C. The absorbance at 450 nm was measured using an ELX800 microplate reader (BioTek Instruments, Inc., USA) to assess cell viability.

### Colony formation

HepG2 or smmc-7721 were planted into 6-well-plate (500 cells/well) and cultivated for 14 days. The growth medium was replenished every two days. Cell colonies were fixed by incubating them in cold methanol at 4° C for a duration of 30 minutes. Subsequently, they were stained using a 1% crystal violet solution in 20% methanol for 30 minutes at 37° C. Then, the staining solution was cleaned and cells were washed with slow running water, followed by natural drying and counting under a light microscope (Nicon; Nikon Corporation, Japan).

### Transwell

Transwell chamber (8-μm pore size; Corning, USA) was used to measure the migration and invasion abilities of hepG2 and smmc-7721cells. For pre-coating, Matrigel was transferred from a temperature of -20° C to 4° C overnight and diluted with serum-free DMEM (at a ratio of 1:5) on ice using a pre-cooled pipette. Afterward, an equal volume of the diluted Matrigel was introduced into the upper transwell chamber and incubated at room temperature for 1 hour. To remove the uncombined Matrigel, the chamber that had been coated was rinsed with serum-free DMEM. Then, 50 μl of serum-free medium with a concentration of 10 g/l of bovine serum albumin (BSA) was added at a temperature of 37° C. In both migration and invasion assays, a total of 5×104 cells were suspended in serum-free DMEM and seeded into the upper chamber. The lower chamber was filled with 500 μL DMEM supplemented with 10% FBS. After incubation for 48 h at 37° C, the migrated or invaded cells were fixed with 4% formaldehyde at room temperature for 20 min and stained with 1% crystal violet (Solarbio, China) for 15 min. Finally, cells were counted under a light microscope (Nicon; Nikon Corporation, Japan).

### Quantitative real time polymerase chain reaction (qRT-PCR)

The TRIzol reagent (AccuRef Scientific, China) was used to extract RNA from cells. According to the manufacturer’s instruction, cDNA was synthesized from total RNA using the PrimeScript® RT Master Mix Perfect Real-Time Reagent kit (Takara Bio, Inc., Japan). With cDNA as the template, The SYBR Green (cat. no. RR420A; Takara Biotechnology ) was used to perform qPCR. in an ABI 7500 Real time PCR instrument (Applied Biosystems, USA) under the following conditions: 95° C for 5 min, 40 cycles of 95° C for 15 s, 58° C for 20 s and 72° C for 10 s. Using GAPDH (mRNA) or U6 (miRNA) as the internal control, the 2-ΔΔCq method was used to calculate fold-changes. The primer sequences of qRT-PCR were tabulated in Table 1.

**Table 1:** Primers for qRT-PCR.

**Gene**	**Primers (5’→3’)**	
	**Forward**	**Reverse**
miR-652-3p	AATGGCGCCACTAGGGTTGTG	Universal PCR Reverse Primer (cat. no. B532451; Sangon Biotech Co., Ltd.)
U6	CTCGCTTCGGCAGCACA	AACGCTTCACGAATTTGCGT
ISL1	TGCCCGCTCCAAGGTGTA	CCGA AGCGCAAATTCGTC
TNRC6A	CTGAGTTTGCCAGTGAAGAG	GCACCATTCCAGTGATTGAG
NPTN	CCAGCTGGACCAATGAAAACC	AGGTCATGTTGCCACCAGTT
CAPZB	CTCCGAGGCCAGCAGA	CGGTCAGTGGGAAGCA
β-actin	ACCGCAAATGCTTCTAGG	ATCCAACCGACTGCTGTC

### Western blotting

The RIPA lysis buffer (AccuRef Scientific, China) was used to extract protein from hepG2 and smmc-7721 cells or exosome. The BCA method (Thermo Fisher Scientific, Inc.) was carried to quantify the extracted protein. After boiling with an equal volume of loading buffer, isovolumetric proteins were separated by 12% SDS-PAGE and then electronically transferred to PVDF membrane (Millipore Sigma, USA). Then, the membrane was blocked with 5% non-fat milk at room temperature for 1h and incubated with indicated anti-CD63 (#ab271286; Abcam, USA), anti-HSP70 (#ab2787; Abcam, USA), anti-TSG101 (#ab125011; Abcam, USA), anti-Calnexin (#ab133615; Abcam, USA), anti-TNRC6A (#SAB2102506; Sigma, USA), and anti-GAPDH (#ab8245; Abcam, USA) primary antibodies overnight at 4° C. For the secondary incubation, membranes were incubated with HRP-conjugated anti-mouse (#97040; Abcam, USA) or anti-rabbit (#7090; Abcam, USA) secondary antibody at room temperature for 1 h. Protein bands were visualized using the Enhanced Chemiluminescence kit (EXINNO, China), ImageJ software (version 1.49, National Institutes of Health) was used to qualify and gray value represented protein amount.

### Dual-luciferase reporter assay

Luciferase reporter gene vector PGL3 and PGL3-TNRC6A contained TNRC6A promoter were purchased from GenePharma (Shanghai, China). Then, reporter gene plasmid was co-transfected with phRL-TK. Cells were cultured in a 24-well-plate until they reached a confluence of 70-80%, then Lipofectamine® 3000 kit (Thermo Fisher Scientific, Inc.) was used to transfect cells with 100 ng pGL3-WT, 20 ng of the transfection control Renilla vector (pRL-TK; Promega Corporation, USA) and 100 nM miR-652-3p mimic or mimic NC (Guangzhou RiboBio Co., Ltd., China). Luciferase activity then was determined: the substrate of Firefly Luciferase LAR II was prepared and dissolved into LAR II buffer, which was stored separately at -80° C and protected from light. 1X PLB was putted in and cells were lysed for 15 min. A substrate of Renilla Luciferase Stop and Glo was used to block the reaction of LAR II. Add 10 ul of cell lysis solution to 40 ul of LAR II, mixing it to test the Firefly Luciferase value. Moreover, add 40 ul Stop and Glo to read the value of Renilla Luciferase. Finally, data processing is carried out: the ratio of firefly luciferase to Renilla luciferase in each tube was calculated, and then the ratio of control group was set to 1 to obtain the relative luciferase activity of each group.

### Statistical analyses

All experiments were performed in triplicate and data are presented as the mean ± standard deviation (SD). The student’s t test was used for comparing between groups. One-way ANOVA with Tukey’s correction for multiple comparisons was used to differentiate data among the multiple groups. P<0.05 was considered statistically significant. Prism 7.0 software (GraphPad, USA) was used to analyze data.

### Availability of data and materials

The datasets used and/or analyzed during the current study available from the corresponding author on reasonable request.
